# Surface modification of PLGA nanoparticles via human serum albumin conjugation for controlled delivery of docetaxel

**DOI:** 10.1186/2008-2231-21-58

**Published:** 2013-07-17

**Authors:** Saeed Manoochehri, Behrad Darvishi, Golnaz Kamalinia, Mohsen Amini, Mahdieh Fallah, Seyed Naser Ostad, Fatemeh Atyabi, Rassoul Dinarvand

**Affiliations:** 1 Department of Pharmaceutics, Faculty of Pharmacy, Tehran University of Medical Sciences, Tehran 1417614411, Iran; 2 Nanotechnology Research Centre, Tehran University of Medical Sciences, Tehran, Iran; 3 Department of Medicinal Chemistry, Faculty of Pharmacy, Tehran University of Medical Sciences, Tehran, Iran; 4 Arash Hospital, Faculty of Medicine, Tehran University of Medical Sciences, Tehran, Iran; 5 Department of Toxicology and Pharmacology, Faculty of Pharmacy, Tehran University of Medical Sciences, Tehran, Iran

**Keywords:** PLGA nanoparticles, Surface modification, Human serum albumin, Emulsion evaporation, Tumor targeting, Docetaxel

## Abstract

**Background:**

Poly lactic-co-glycolic acid (PLGA) based nanoparticles are considered to be a promising drug carrier in tumor targeting but suffer from the high level of opsonization by reticuloendothelial system due to their hydrophobic structure. As a result surface modification of these nanoparticles has been widely studied as an essential step in their development. Among various surface modifications, human serum albumin (HSA) possesses advantages including small size, hydrophilic surface and accumulation in leaky vasculature of tumors through passive targeting and a probable active transport into tumor tissues.

**Methods:**

PLGA nanoparticles of docetaxel were prepared by emulsification evaporation method and were surface conjugated with human serum albumin. Fourier transform infrared spectrum was used to confirm the conjugation reaction where nuclear magnetic resonance was utilized for conjugation ratio determination. In addition, transmission electron microscopy showed two different contrast media in conjugated nanoparticles. Furthermore, cytotoxicity of free docetaxel, unconjugated and conjugated PLGA nanoparticles was studied in HepG2 cells.

**Results:**

Size, zeta potential and drug loading of PLGA nanoparticles were about 199 nm, −11.07 mV, and 4%, respectively where size, zeta potential and drug loading of conjugated nanoparticles were found to be 204 nm, −5.6 mV and 3.6% respectively. Conjugated nanoparticles represented a three-phasic release pattern with a 20% burst effect for docetaxel on the first day. Cytotoxicity experiment showed that the IC50 of HSA conjugated PLGA nanoparticles (5.4 μg) was significantly lower than both free docetaxel (20.2 μg) and unconjugated PLGA nanoparticles (6.2 μg).

**Conclusion:**

In conclusion surface modification of PLGA nanoparticles through HSA conjugation results in more cytotoxicity against tumor cell lines compared with free docetaxel and unconjugated PLGA nanoparticles. Albumin conjugated PLGA nanoparticles may represent a promising drug delivery system in cancer therapy.

## Background

Polymeric nanoparticles are considered as one of the most promising carrier systems for cytotoxic drug delivery and have received a great deal of attention during the recent years. Poly lactic-co-glycolic acid (PLGA) based nanoparticulate system is one of the most successful and interesting colloidal systems. PLGA nanoparticles (NPs) protect the therapeutic agents and increase their stability and can be used for controlled delivery of therapeutics with improved pharmacokinetic and pharmacodynamic profile. Furthermore, PLGA is approved by United States Food and Drug Administration (FDA) and European Medicine Agency (EMA) for various drug delivery formulations. However, PLGA NPs, suffer from a significant limitation due to their high level of opsonization by reticuloendothelial system (RES) [1]. To address this negative aspect, several methods and procedures have been utilized for surface modification of PLGA NPs in order to produce PLGA based nanoparticulate systems which are not readily recognized by RES. This goal has been achieved by PLGA NPs surface coating with more hydrophilic agents to cover their hydrophobic surface and provide stealth nanoparticles. Various agents have been used for this purpose including polyethylene glycol (PEG), poloxamers and chitosan molecules to neutralize or reduce the negative zeta potential of PLGA NPs and reduce their phagocytosis by RES [2]. However, surface modified PLGA NPs have been found to highly localize in liver tissue due to the rigidity of the PLGA core which has triggered many research studies to improve surface modification methods for achieving better outcomes [3,4].

Among various available agents, human serum albumin (HSA) seems to be a promising molecule for surface modification of PLGA NPs. HSA is known to be the most abundant native protein in human body which has various advantages including ready availability, biodegradability, and low toxicity and immunogenicity [5]. HSA has received special interest in drug delivery studies due to its ability for passive targeting through enhanced permeation and retention (EPR) effect which is related to both “enhanced permeation” of macromolecules through the fenestrated structure of tumor vasculature in addition to an “enhanced retention” which is considered to be related to the lack of effective lymphatic drainage in tumor tissues [6]. Furthermore it has been postulated that active targeting of HSA coated nanoparticles occurs through special albumin receptors on tumor cell membranes [7].

Surface modification of PLGA NPs by grafting protein molecules has been previously reported and various proteins including transferrin and wheat germ agglutinin have been coated on the surface of PLGA NPs through covalent conjugation. The created stealth NPs have successfully increased the cellular uptake of PLGA NPs and have resulted in a greater antiproliferative activity and better cytotoxicity in vitro. In addition in vivo experiments have confirmed a higher tumor accumulation for the above surface coated NPs [8-10]. HSA coating by non-covalent interactions have also been studied where protein molecules only saturate the nanoparticles surface and no covalent linking occurs [11].

Herein, docetaxel (DTX), a cytostatic agent with very low water solubility was encapsulated in PLGA NPs produced by a modified emulsification evaporation method and was subjected to surface modification by HSA molecules through a carbodiimide conjugation reaction. Surface modification created a stealth core shell structure and was used to reduce the negative charge on PLGA NPs along with an increase in drug efficiency through passive targeting. These newly developed fully biodegradable HSA coated PLGA NPs can increase DTX water solubility and will eliminate the need for Tween 80 utilization in DTX formulations which has been taken responsible for many of DTX injections side effects including hypersensitivity reactions [12]. The nanoparticles were further evaluated for their cytotoxic effect in HepG2 cell line. The present evidences suggest that HSA stealth PLGA NPs may represent a promising nanoparticulate drug delivery system for DTX and other anticancer agents.

## Methods

### Materials

PLGA (50:50, 504H, MW = 48 kDa) was purchased from Boehringer-Ingelheim (Ingelheim, Germany). HSA, 1-ethy1-3-(3-dimethylaminopropyl) carbodiimide (EDC), N–hydroxysuccinimide (NHS) and 3-(4,5-dimethyathiazol-2-yl)-2,5-diphenyltetrazoliumbromide (MTT) were purchased from Sigma-Aldrich (St. Louis, MO, USA). Dulbecco’s modified eagle’s medium (DMEM), fetal bovine serum (FBS) and penicillin and streptomycin antibiotic mixture were obtained from Life technologies (grand Island, NY, USA).Polyvinyl alcohol (PVA) was obtained from Acros (Geel, Belgium). 2-(N-morpholinoethansulfonicacid) (MES) was purchased from Fluka (St. Louis, MO, USA). DTX was from Cipla (Mumbai, India). All other solvents and reagents unless otherwise stated were from Merck (Darmstadt, Germany).

### Preparation of PLGA nanoparticles

PLGA NPs were fabricated using a modified emulsification/ evaporation method [12]. Briefly, PVA (0.5% w/w) was dissolved in deionized water providing the aqueous phase where MES buffer (50 mmol) was used for pH adjustment (pH = 5). In organic phase, PLGA and docetaxel were dissolved in mixtures with various ratios of acetone and dichloromethane and the resulting organic phase was slowly added to the aqueous phase using an ultrasonic probe (Misonix, USA) equipped with a microtip (power: 11 watt, time: 5 min). During sonication, the whole system was placed in an ice bath to prevent any loss of the oil phase due to evaporation and the mixture was magnetically stirred overnight. The experiment was conducted with 10% and 20% organic phase to water phase ratio to evaluate the effect of oil: water ratio on the formed nanoparticles characteristics. Purification of nanoparticles was achieved by three times centrifugation with a high speed centrifuge (21,000 g for 30 minutes) at 10°C (Sigma 3 K30, Ostrode, Germany) to remove any extra PVA from the NPs surface. The isolated NPs were then lyophilized into a fine cake.

### Surface modification of PLGA NPs by HSA conjugation

PLGA NPs were conjugated with HSA through a carbodiimide coupling reaction. For this purpose, PLGA carboxylic functional groups were first activated through exposure with 15 mg EDC and 12 mg NHS for 5 h right after their preparation in a medium consisting of MES buffer (pH = 5, 50 mmol). The activated NPs were then purified by three times centrifugation (21,000 g for 30 minutes) at 10°C to remove any remnants of PVA, EDC and NHS.

The activated PLGA nanoparticles were then exposed to HSA solution in 40 ml phosphate buffered saline (PBS, pH = 7.4) with an equimolar concentration in a dropwise manner and under constant stirring. The conjugated NPs were then isolated by a centrifugation step (15,000 g for 30 minutes) at 10°C and the supernatant was removed. Conjugated NPs were then subjected to the lyophilization process for long term storage.

### Determination of HSA conjugated PLGA NPs molecular structure

For determining the number of free carboxylic moieties in activated PLGA NPs and further calculation of the activation ratio a Bruker 500 MHz ^1^H-NMR system (Bruker, Germany) was used and the resulting nuclear magnetic resonance (NMR) spectrum was utilized to characterize the substitution degree of activated PLGA.

Fourier transform infrared (FT-IR) spectroscopy was used to characterize the molecular structure of PLGA NPs conjugated with HSA and to confirm the structural changes occurred after HSA conjugation. Lyophilized samples were placed on KBr disks and their FT-IR spectra were recorded by a Nicolet (Magna IR-550, USA) spectrophotometer.

### NPs characterization and morphology evaluation

PLGA and PLGA NPs conjugated with HSA were fully characterized and their size, poly dispersity index (PDI) and zeta potential were determined by dynamic light scattering (DLS) (Malvern Zetasizer, ZS, Malvern, UK).

To evaluate the particles morphology, PLGA NPs were coated with gold under vacuum conditions and their morphology was evaluated by scanning electron microscopy (SEM) (FE-SEM, S4160, Hitachi, Japan).

Furthermore, transmission electron microscope (TEM) (Zeiss EM10C, Carl Zeiss AG, Oberkochen, Germany) was used to evaluate the surface morphology of PLGA NPs conjugated with HSA.

Differential scanning calorimetry (DSC) was performed for PLGA NPs conjugated with HSA in addition to a physical mixture of PLGA, HSA and DTX with a Mettler DSC 823 unit (Mettler Toledo, Switzerland) and a Julabo thermocryostate model FT100Y (Julabo labortechnik, Germany) where Indium was utilized for instrument calibration. The scanning took place between 20 to 200°C with a thermal scan rate of 10°C per minute.

### Determination of DTX concentration, drug loading and entrapment efficiency in nanoparticles

The extent of DTX loading in PLGA and PLGA NPs conjugated with HSA was investigated by a previously published method [13]. Briefly, a small amount of PLGA NPs (2 mg) was dispersed in 2 ml acetonitrile resulting in PLGA and DTX dissolution in acetonitrile medium. In the next step methanol was added to the above mixture and resulted in PLGA precipitation. This solution only contained DTX and was injected to high pressure liquid chromatography (HPLC) (Knauer apparatus model K-1001 Wellchrome (Berlin, Germany) equipped with a PDA K-2700 ultraviolet Detector (Knauer, Berlin, Germany). A C18 column (internal diameter: 0.46 cm, length: 25 cm, pore size: 5 μm, ODS 3-MZ, Germany) was used as stationary phase and acetonitrile: water mixture (65: 35) was used as the mobile phase. The analysis was performed in 230 nm wavelength at 25°C and DTX concentration was evaluated accordingly. Furthermore, “drug loading” which is calculated as the ratio between DTX (expressed as μg) and the PLGA NPs (expressed as mg) in addition to “entrapment efficiency” or the percent of drug loaded in PLGA nanoparticles to the initial amount of DTX were determined.

### DTX release from PLGA nanoparticles

Dialysis technique was used to evaluate drug release from PLGA NPs [14]. A dialysis tube (molecular weight cut-off: 8 kDa) containing 2 mg of nanoparticles suspended in 5 ml PBS (pH = 7.4) was dipped in a medium consisting of 20 ml PBS (pH =7.4) containing 0.1% Tween 80 and was continuously stirred on a shaker incubator for over 14 days at 37°C. At selected time intervals, 15 mL of the medium was removed and the medium was replenished with freshly prepared PBS. The aliquots were then exposed to dichloromethane for liquid-liquid extraction of DTX. The extraction procedure was repeated three times and the extracted organic phase was evaporated and the remnants were dissolved in 200 μl acetonitrile and were subjected to HPLC analysis. Drug release data were expressed as the percentage of the cumulative amount of drug release.

### In vitro cytotoxicity of DTX encapsulated NPs

HepG2 (Human hepatocellular carcinoma) cells were obtained from Pasteur Institute of Iran (Tehran, Iran) and were cultivated and maintained in DMEM growth media supplemented with 10% FBS and 1% penicillin streptomycin antibiotic mixture. The cells were incubated at 37°C in a humidified atmosphere with 5% CO_2_ and the culture media was exchanged every other day. Cells were seeded in 96 wells with a density of 1 × 10^4^ cells per well and were incubated for 24 h to allow cellular attachment. Cells were then treated with different concentrations of free DTX,PLGA NPs and PLGA NPs conjugated with HSA for 96 hand were then exposed to 20 μL of MTT reagent (5 mg/mL per well) for an additional 4 h. The culture media was then discarded and 200 μL dimethylsulfoxide was added to each well to dissolve the formed formazan crystals. Optical density was then measured at 570 nm with a 660 nm background using a microplate reader (Anthos 2020; Anthos Labtec Instruments, Wals, Austria). Each experiment was performed in triplicate and cellular viability was expressed as the percentage of viable cells to control. IC50 was calculated from the cell viability data as the drug concentration in which cell growth was inhibited by 50%. Furthermore similar MTT assays were performed with drug free PLGA NPs and conjugated PLGA NPs with HSA to assess any intrinsic carrier toxicity.

## Results and Discussion

### PLGA nanoparticles preparation and characterization

**Figure 1:**
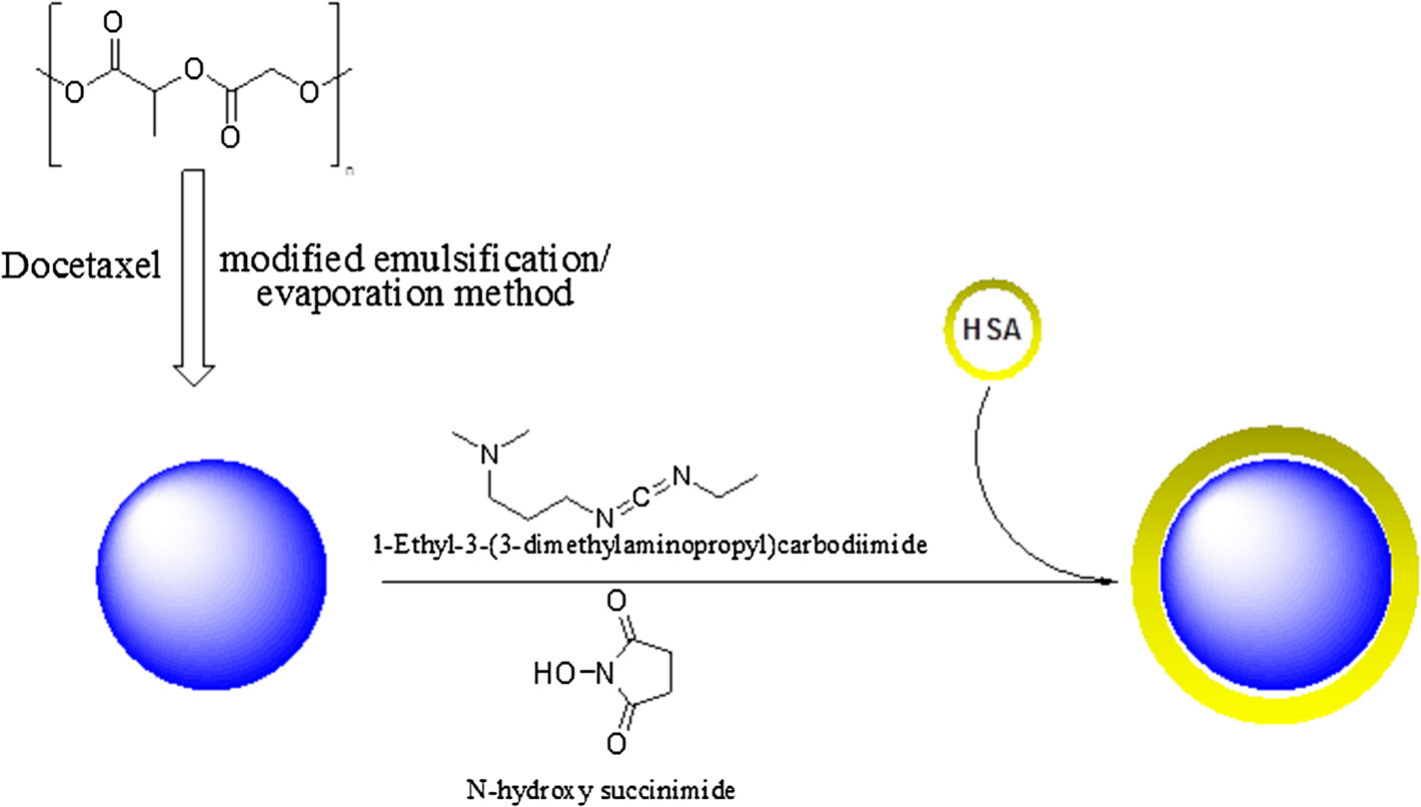
PLGA and conjugated PLGA nanoparticles with HSA formation process.

PLGA nanoparticles were prepared by a modified emulsification evaporation method (Figure 1). In this method the organic phase solution containing DTX and PLGA provides the oil phase (O) and PVA aqueous solution serves as the water phase (W) in an O/W emulsion. Physical sonication induction reduces the size of nano droplets in this emulsion. The particulate characteristics of prepared PLGA NPs in addition to their drug loading and entrapment efficiency are shown in Table 1. As it is observed PLGA NPs had an average size of 180 to 221 nm and were monodispersed as confirmed by their PDI amounts which was less than or equal to 0.23. Nanoparticles drug loading was achieved by drug incorporation into PLGA NPs during the production process and a maximum 5.35% drug loading with about 48% entrapment efficiency was achieved. The formed NPs carried a negatively charged zeta potential of about -11mv.

**Table 1:** PLGA nanoparticles and PLGA nanoparticles conjugated with HSA characteristics (DCM: dichloromethane, O: oil phase, W: water phase, HSA: human serum albumin, PLGA: Poly (lactic-co-glycolic acid), PDI: polydispersity index and EE: entrapment efficiency)

**Sample**	**Acetone: DCM**	**O:W ratio (%)**	**HSA:PLGA**	**Size (nm)**	**PDI**	**Zeta potential (mv)**	**E.E (%)**	**Loading (μg/mg)**
1	2.5:97.5	20	-	221 ± 25	0.23 ± 0.02	−13.48 ± 1.8	18.5 ± 2.1	2.06 ± 0.18
2	10:90	20	-	195 ± 19	0.15 ± 0.01	−11.71 ± 2.1	25.2 ± 1.8	2.8 ± 0.41
3	20:80	20	-	188 ± 28	0.14 ± 0.01	−15.32 ± 2.3	9.3 ± 1.6	1.0 ± 0.2
4	40:60	20	-	180 ± 29	0.20 ± 0.02	−11.13 ± 1.9	6.2 ± 1.1	0.7 ± 0.05
5	5:95	10	-	204 ± 20	0.15 ± 0.02	−11.52 ± 1.5	13.5 ± 1.1	1.5 ± 0.1
6	10:90	10	-	199 ± 22	0.17 ± 0.01	−11.07 ± 1.8	48.2 ± 2.3	5.35 ± 0.33
7	20:80	10	-	189 ± 19	0.18 ± 0.02	−9.83 ± 1.3	9.46 ± 1.4	1.05 ± 0.07
8	30:70	10	-	186 ± 22	0.16 ± 0.01	−11.08 ± 1.4	2.8 ± 0.7	0.3 ± 0.03
9	40:60	10	-	180 ± 21	0.20 ± 0.02	−11.02 ± 1.7	7.6 ± 1.5	0.85 ± 0.1
10	10:90	10	50:50	204 ± 15	0.14 ± 0.01	−5.6 ± 0.21	33.7 ± 2.6	3.62 ± 0.42
11	10:90	10	75:25	221 ± 24	0.17 ± 0.01	−5.45 ± 0.24	34.2 ± 2.1	3.53 ± 0.37

Size is the most important and effective parameter in determining the cellular uptake and biological functions of colloidal systems where a reduced size will usually result in significantly better uptake of nanoparticles by tumor cells [15]. It has been demonstrated that emulsion fabrication is the most critical step during the preparation of nanoparticles where the size of emulsion droplets is directly relevant to the final nanoparticles size [16,17]. Various factors may affect the size of PLGA NPs including the stabilizer type and the oil phase solvent mixture composition.

**Figure 2:**
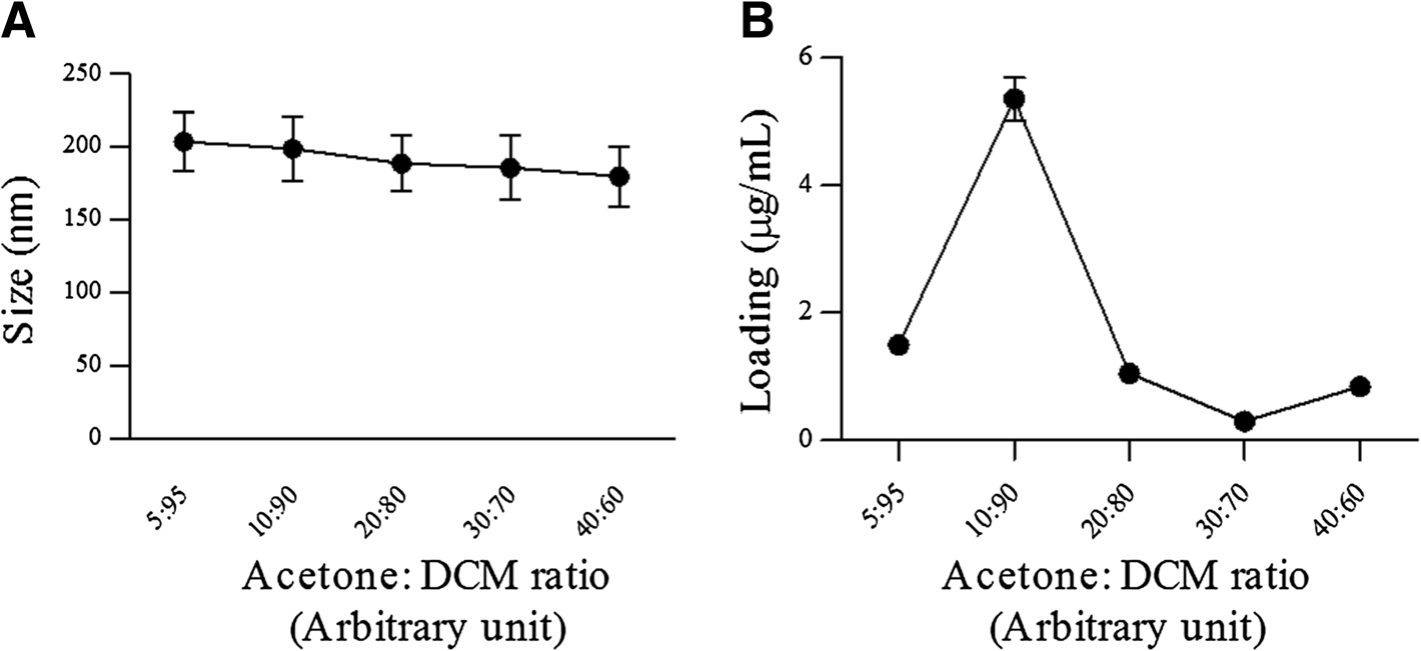
The effect of acetone to dichloromethane ratio on PLGA nanoparticles size (A) and drug loading (B).

The oil phase solvent mixture here was composed of various ratios of dichloromethane and acetone. As shown in Figure 2A, nanoparticles size decreased as a result of reducing the acetone to dichloromethane ratio in the oil phase mixture where the smallest size was achieved by a 40:60 (acetone: dichloromethane) ratio. This may be due to a decrease in the interfacial tension between the oil phase and aqueous phase with increasing the portion of acetone in the oil phase mixture. It has been reported that during the emulsification process the two organic and aqueous phases are not in equilibrium and the acetone concentration is not homogenous at the interface of oil and water phases. Acetone diffuses through the higher viscosity oil phase resulting in an acetone concentration gradient which creates some perturbation and turbulence in the interface of the two phases. This perturbation further results in a larger interface area between the oil droplets and aqueous medium and creates extremely fine nano droplets which result in the formation of small sized nanoparticles. The interfacial tension decrease between the oil and water phases due to the presence of acetone in the organic solvent mixture in addition to acetone passive diffusion from nano droplets and the subsequent spontaneous mixing will result in PLGA NPs formation with a much smaller size [17].

Besides the size of NPs, loading and entrapment efficiency are also affected by acetone to dichloromethane ratio. As it is observed in Figure 2B, loading is decreased with increasing the acetone: dichloromethane ratio from 10:90 to 40:60. In addition entrapment efficiency shows the same trend and a decrease in this parameter is observed with increasing acetone to dichloromethane ratio. DTX has poor water solubility and favors the organic phase during oil in water emulsion preparation. This higher drug affinity to the oil core results in a higher drug loading and better entrapment efficiency [18]. However when acetone to dichloromethane ratio is very low, both loading and entrapment efficiency are significantly decreased which may be related to the high DTX solubility in acetone solutions [19]. On the other hand when acetone to dichloromethane ratio is increased, some of the drug molecules dissolved in the oil core may disperse out along with acetone. This results in some sort of drug leak and will decrease the drug loading [17].

In addition to the oil phase composition, the ratio of oil to water is another factor which may impact the evaporation phase and nanoparticles formation process. Decreasing the oil to water ratio from 20% to 10% has mildly increased the nanoparticles size along with a slight increase in their entrapment efficiency and drug loading (Table 1). It has been postulated that when the aqueous phase volume is increased in relation to oil phase, the droplets external energy which is the cause of droplets breakage is distributed in a higher volume and a lower droplet breakage and an elevated particle size will be achieved [20,21].

Nanoparticles stabilization and protection of the droplets is another important step in the emulsification process which is achieved by addition of surfactant molecules. These surfactants prevent drops coalescence and result in a better size. In this study 0.5% PVA was used as a stabilizer to create a cover between organic and aqueous phases interface and provide effective stabilization [22,23].

**Figure 3:**
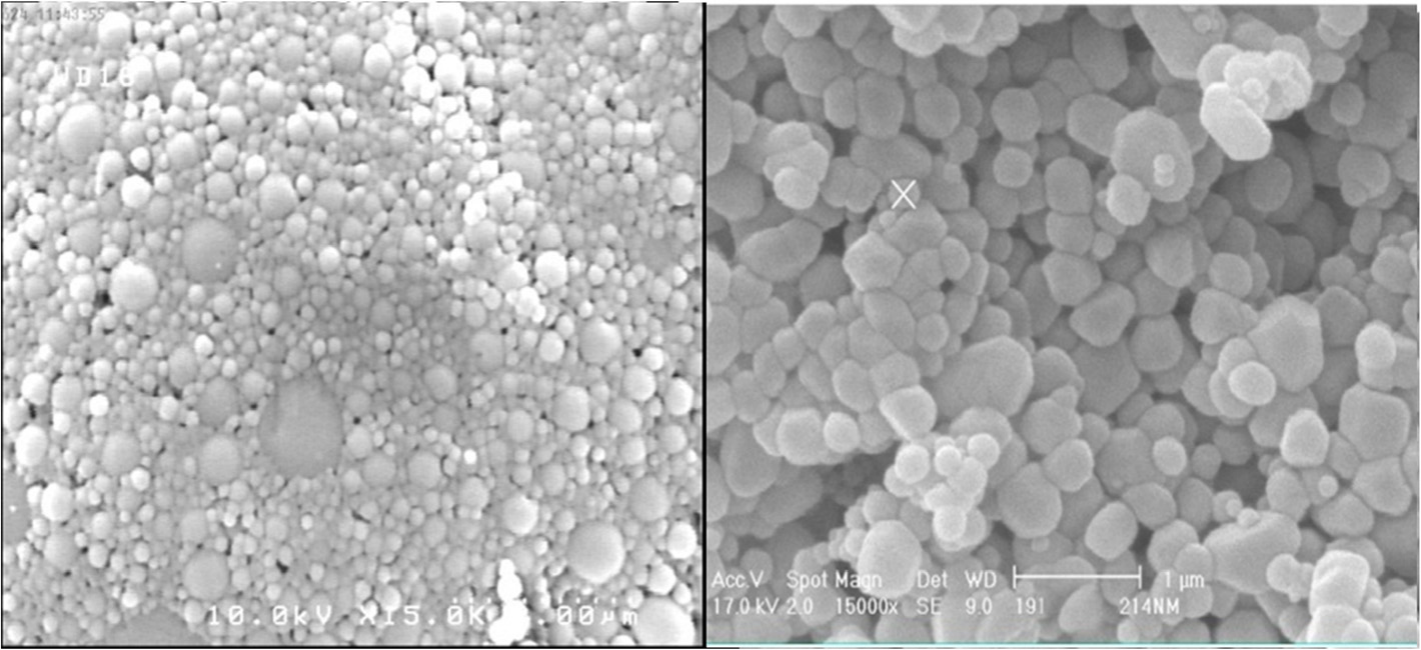
Poly (lactic-co-glycolic acid) (PLGA) nanoparticles morphology acquired by scanning electron microscopy with 15,000 times magnification.

PLGA NPs size and morphology was further evaluated by SEM microscopy which showed roughly spherical particles (Figure 3). It is noteworthy that NPs showed a larger size when evaluated by DLS compared to SEM images which may be due to the swelling of particles when dispersed in the aqueous medium [16].

### Characterization of PLGA nanoparticles conjugated with HSA

**Figure 4:**
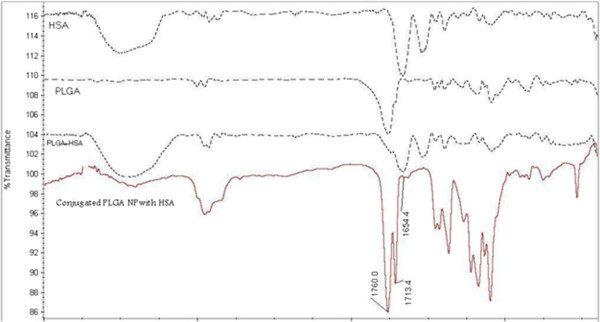
**FT-IR spectrum of HSA, PLGA, their physical mixture and PLGA NPs conjugated with HSA (PLGA major absorption peaks are observed in 1760 (cm**^**-1**^**) and 1713 (cm**^**-1**^**) where HSA major absorption peak comes in 1650 (cm**^**-1**^**)).**

HSA was conjugated to carboxylic functional groups on PLGA structure through a carbodiimide meditated coupling reaction Figure 1). FT-IR spectra of PLGA, HSA, their physical mixture and PLGA NPs conjugated with HSA are shown in Figure 4. As it can be seen, free PLGA ester and acid bonds absorption peaks can be observed in 1760 (cm^-1^) and 1713 (cm^-1^). In free HSA the amide bond stretch region was observed in 1650 (cm^-1^). When HSA conjugated PLGA nanoparticles spectrum was evaluated, 1760 (cm^-1^), 1713 (cm^-1^) and 1650 (cm^-1^) absorption peaks were observed which confirms the presence of both PLGA and HSA structures in the conjugated NPs.

Activated PLGA NPs structure was further evaluated by its ^1^H NMR spectra to predict the ratio of covalently conjugated carboxylic functional groups on PLGA NPs. For this purpose area under the curve of NHS peak at 2.0 ppm was compared with area under the curve of PLGA peaks at 5.4, 4.9 and 1.5 ppm in activated PLGA NMR spectra. It was found that about 30% of PLGA carboxylic acid moieties were involved in PLGA activation reaction where about 70% of carboxylic functional groups remained free. This suggests a 30% conjugation ratio between PLGA and HSA.

To further evaluate HSA conjugated PLGA nanoparticles, size and zeta potential of these particles were evaluated and were compared with unconjugated PLGA NPs (Table 1). As it is observed, the size of PLGA NPs conjugated with HSA was slightly increased in comparison to PLGA NPs.

Zeta potential which is another important nanoparticle characteristic and can influence particle stability and cell adhesion was also measured in PLGA NPs conjugated with HSA [24]. As shown in Table 1, the zeta potential of PLGA NPs conjugated with HSA showed a negative zeta potential, which was smaller than that of PLGA NPs (−11.07 ± 1.8 mV vs. -5.6 ± 0.21 mV). This decrease in negative zeta potential is probably due to the involvement of carboxylic functional groups on PLGA NPs in amide bond formation with amino moieties on lysine amino acids in HSA primary structure [25].

Entrapment efficiency and loading of PLGA NPs and PLGA NPs conjugated with HSA are shown in Table 1. As it is shown, the entrapment efficiency and drug loading in PLGA NPs conjugated with HSA were slightly less than PLGA NPs.

**Figure 5:**
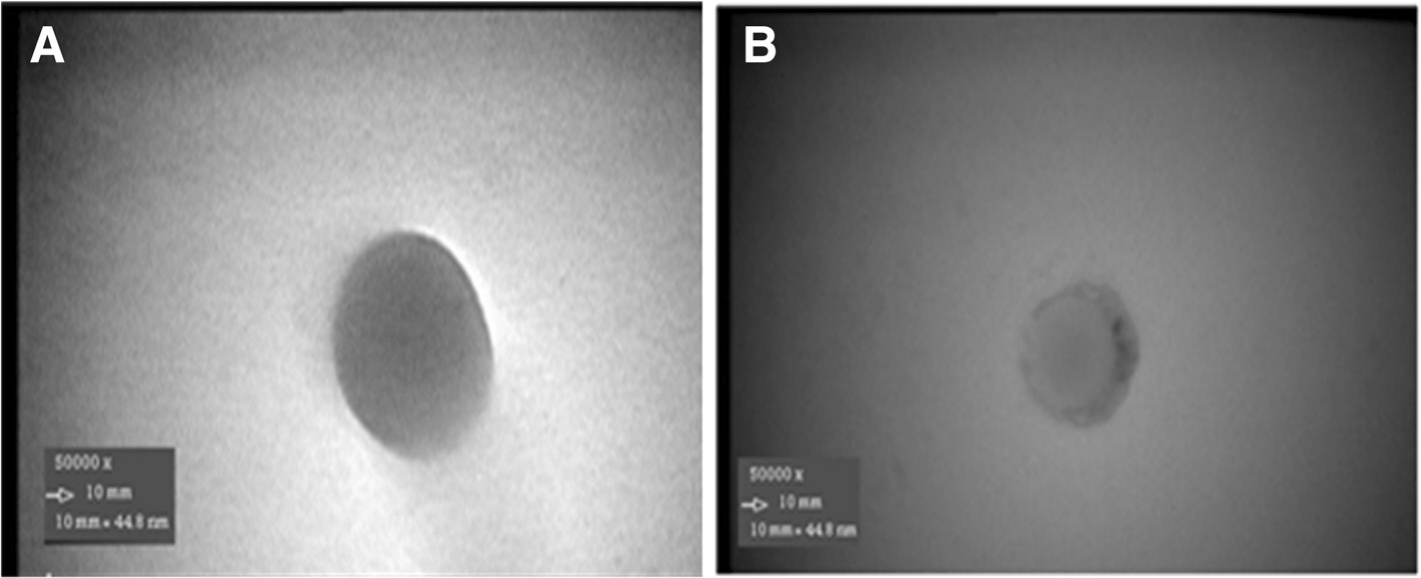
Appearance of A) PLGA nanoparticle and B) PLGA nanoparticle conjugated with HSA acquired by transmission electron microscopy with 50,000 times magnification (Conjugation has occurred in the surface of PLGA nanoparticles where phase contrast is clearly observe).

PLGA NPs morphology was evaluated by TEM imaging (Figure 5). As it is observed PLGA NPs were almost spherical with a size of about 200 nm where HSA conjugated NPs showed a slightly larger size. PLGA NPs were uniform but a notable outer layer was observed in conjugated NPs. It seems that HSA, have formed a mushroom structure on NPs surface which is in accordance with the predicted core-shell structure and may be able to prevent PLGA NPs opsonization more effectively [26].

Drug polymer mixtures may exist in various forms and structures for example amorphous or crystalline drug in combination with an amorphous or crystalline polymer [27]. DSC was utilized here to examine the thermal transitions in PLGA NPs and PLGA NPs conjugated with HSA.

**Figure 6:**
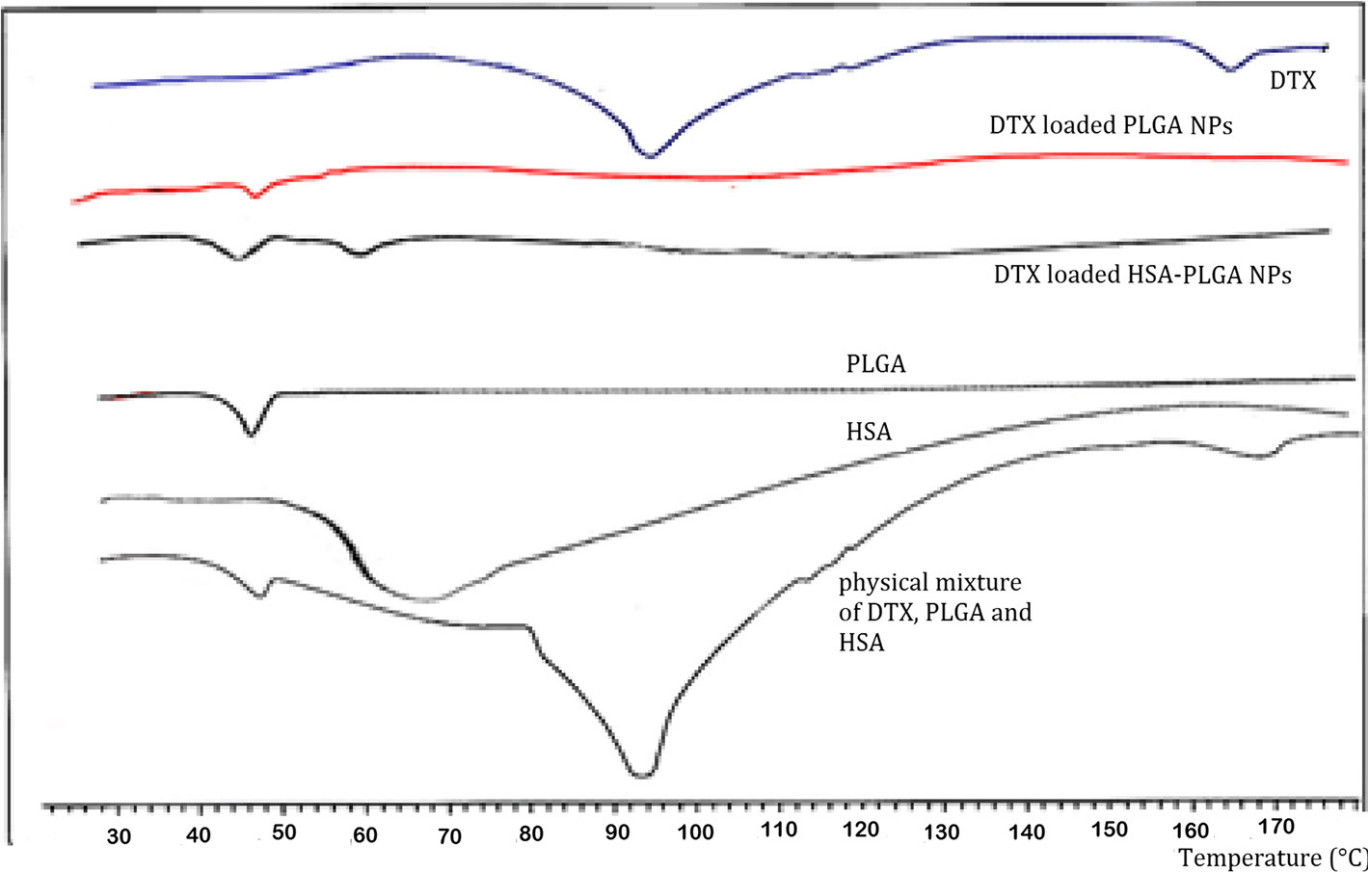
**DSC thermograms of PLGA, HSA, DTX and DTX loaded in PLGA NP conjugated with HSA (Peaks of PLGA, HSA, and DTX was observed at 48°C, 60°C and 160°C.** In DTX encapsulated HSA-PLGA nanoparticles, PLGA peak was observed at 48°C, HSA peak was observed at 60°C and no peak was observed at 160°C indicating the amorphous structure of DTX in this nanoparticulate formulation).

Figure 6 shows DSC thermograms of PLGA, DTX, HSA, PLGA NPs and PLGA NPs conjugated with HSA in addition to a physical mixture of PLGA, DTX and HSA.As it can be seen, PLGA has an endothermic glassy transition temperature around 50°C. Glassy transition endothermic process for HSA conjugated NPs was observed at about 60°C. In the physical mixture thermogram a peak is observed in 160°C which stands for DTX melting point peak in its crystalline form. This peak is not observed in PLGA NPs and PLGA NPs conjugated with HSA which suggests an absence of crystallinity and an amorphous structure for DTX molecules in these NPs [28,29].

### DTX release pattern from PLGA and HSA conjugated PLGA NPs

**Figure 7:**
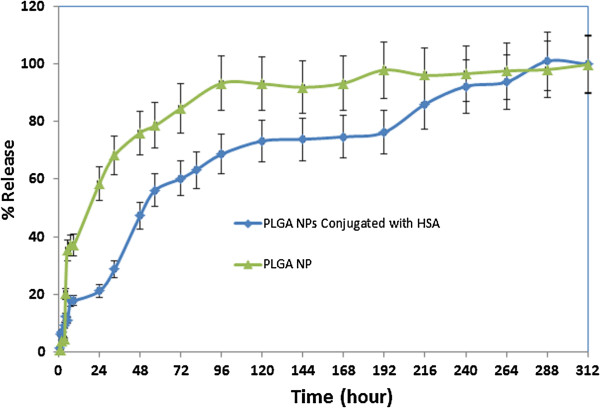
**DTX release pattern from PLGA NPs and PLGA NPs conjugated with HSA in PBS medium (pH = 7.4).** In PLGA NPs, 40% burst release of DTX occurred in the first 10 hours. The drug was then released slowly within 12 days (green line); In PLGA NPs conjugated with HSA, 20% burst release of DTX occurred within 24 hours and the overall release rate was much slower compared to PLGA NPs (blue line).

Various factors may affect drug release behavior from PLGA NPs including the polymer type and composition and drug-polymer interactions. In vitro drug release pattern from PLGA and PLGA NPs conjugated with HSA is shown as the cumulative percentage of release in Figure 7. DTX release from PLGA NPs followed a bi-phasic pattern. As shown in Figure 7, a burst release occurs within 10 h when roughly 40% of the drug was released from the nanoparticles. The rest of DTX was released with a slower rate within 12 days. This initial burst release may be related to the portion of DTX molecules which are adsorbed on nanoparticle’s surface and the diffusion controlled drug dissolution of drug molecules at nanoparticles and medium interface [30]. In addition the release of DTX molecules which are encapsulated near the surface of PLGA NPs may also contribute in this initial burst release. The slower second phase is related to the entrapped DTX inside the polymeric NPs.

The release profile of DTX from PLGA NPs conjugated with HSA exhibited a three-phasic pattern (Figure 7). 20% initial burst release occurred at the first day and in the next 8 days 75% of DTX was released. The rest of the drug was eventually released within 4 days. Comparing the release pattern of these two nanoparticles demonstrated that the release rate of DTX from conjugated NPs was slower and followed a three phasic pattern including an initial burst phase, a second release phase and a much slower final drug release phase.

The graph shows that surface treatment with HSA molecules has resulted in a reduced initial burst where only 20% of drug is released during the first 24 h from HSA conjugated NPs in comparison to around 60% in unconjugated NPs. This lower burst effect is probably due to a decrease in the amount of drug which is adsorbed on the surface of conjugated NPs [9].

### In vitro cytotoxicity assay

**Figure 8:**
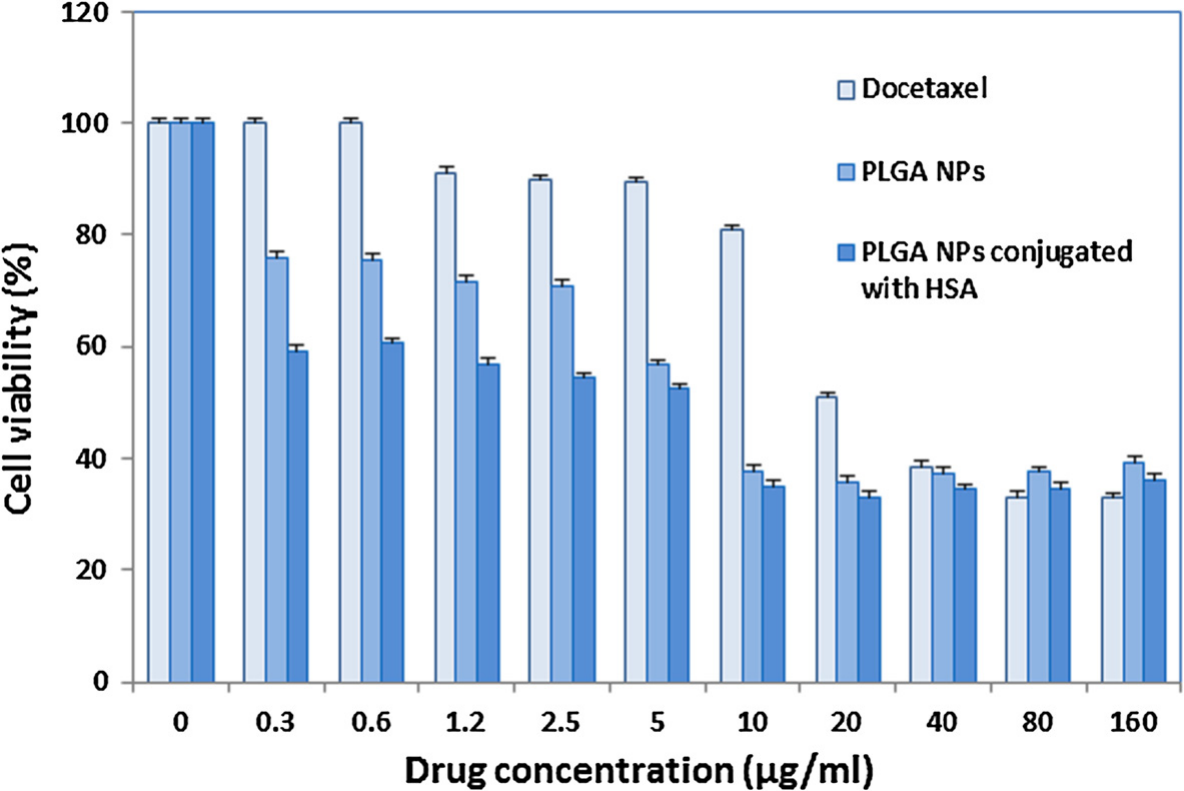
Cell viability in HepG2 cell line in exposure to free DTX, DTX encapsulated PLGA NPs and DTX encapsulated HSA-PLG NPs with various concentrations against Hep-G2 assessed by MTT assay (n = 3).

To assess the cellular cytotoxicity of PLGA NPs and PLGA NPs conjugated with HSA, HepG2 cell line was used. HepG2 cell line has been recognized as a suitable model for cytotoxicity evaluating studies [31]. Following 72 h of exposure to both unconjugated and conjugated NPs, cell viability was assessed by MTT assay and IC50 values of free DTX and its formulations were estimated from the results as presented in Figure 8. The IC50 of free DTX, PLGA NPs and PLGA NPs conjugated with HSA were 20.2 ± 0.28, 6.2 ± 0.23 and 5.4 ± 0.21 μg respectively after 72 h incubation with the cell line (n = 3). These results may be explained by drug release pattern of various PLGA particulate formulations and their degradation profiles. When DTX is encapsulated in PLGA or PLGA NPs conjugated with HSA a higher DTX concentration may be delivered to the intracellular space which may be the result of the small size of nanoparticles and the passive targeting of macromolecules due to EPR effect. As a result a better efficacy and a lower IC50 is observed for HSA conjugated with PLGA NPs [32].

It should be mentioned that in PLGA NPs conjugated with HSA, the outer layer, HSA, creates a stealth surface which reduces NPs interaction (both interactions between NPs and interaction between cells and NPs) [33].

It is noteworthy that no cellular cytotoxic effect was observed when drug free PLGA NPs and drug free PLGA NPs conjugated with HSA were exposed to HepG2 cell line which confirms these carriers safety.

## Conclusions

In this study surface modified PLGA NPs with HSA were developed to prepare a novel drug delivery particulate system which may preferentially target tumor tissues through passive targeting. Specific features of HSA such as its biocompatibility and accumulation in inflamed and tumor tissues make it a good candidate for surface functionalization of carriers. Herein, PLGA NPs conjugated with HSA were developed and fully characterized. The newly developed NPs had an acceptable size and zeta potential and showed promising results in cell culture investigation where a lower IC50 was observed for the conjugated nanoparticles in comparison with free DTX and unconjugated PLGA NPs. It seems that surface modification of PLGA NPs with human serum albumin may represent a promising approach for nanoparticulate delivery system for cytostatic agent delivery.

## Competing interests

The authors declare that they have no competing interests.

## Authors’ contributions

SM conceived the study, carried out the experiments and drafted the manuscript. BD helped with the characterization of the nanoparticles, GK reviewed and revised the manuscript. MA supervised the synthesis and characterization of copolymers, MF assisted in preparation of the manuscript, SNO supervised the MTT assay and cell culture study, FA co-supervised the study, RD designed, coordinated and supervised the study and is the corresponding author of the manuscript. All authors read and approved the final manuscript.
